# Supplementary material for: Control of cell morphology and differentiation by substrates with independently tunable elasticity and viscous dissipation
Source: Nat Commun. 2018 Jan 31;9:449. doi: 10.1038/s41467-018-02906-9 (PMC5792430; doi:10.1038/s41467-018-02906-9)
Supplement: Supplementary file 1 — Supplementary Information [file 41467_2018_2906_MOESM1_ESM.pdf]

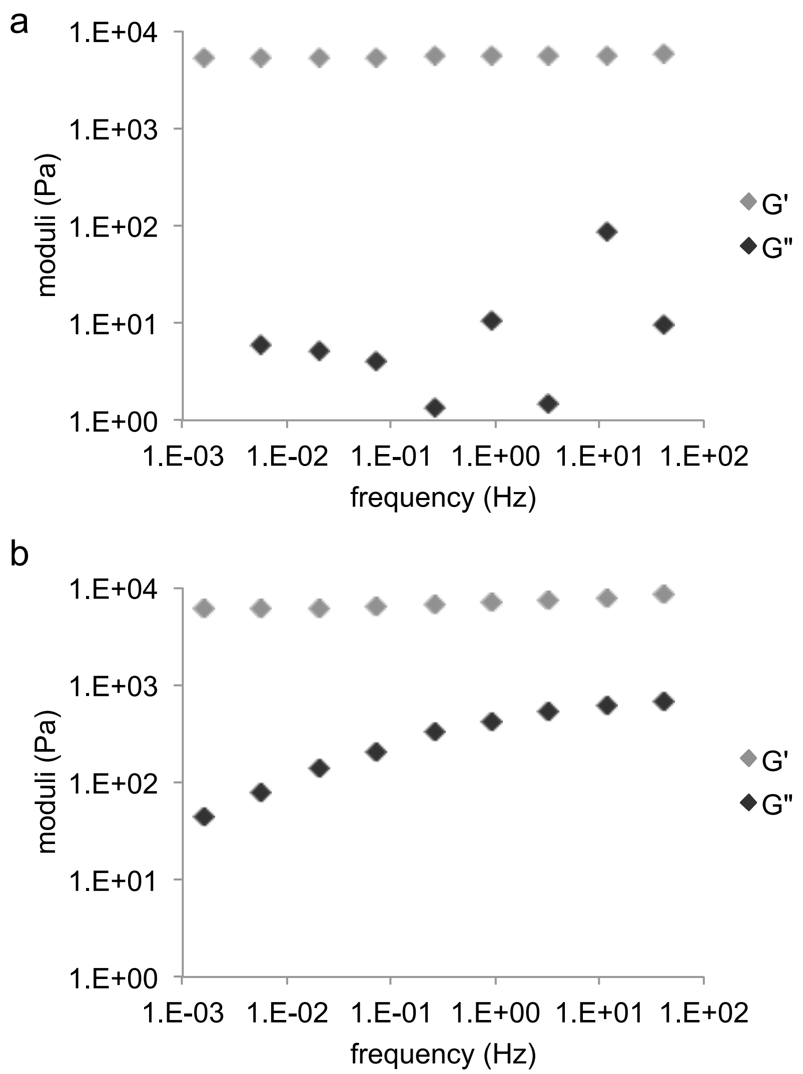

Supplementary Figure 1: Frequency sweep analysis of 5kPa gels

a: Frequency dependence of G’ and G” of a purely elastic PAA gel.

b: Frequency dependence of G’ and G” of a PAA gel containing 1.8% linear acrylamide.

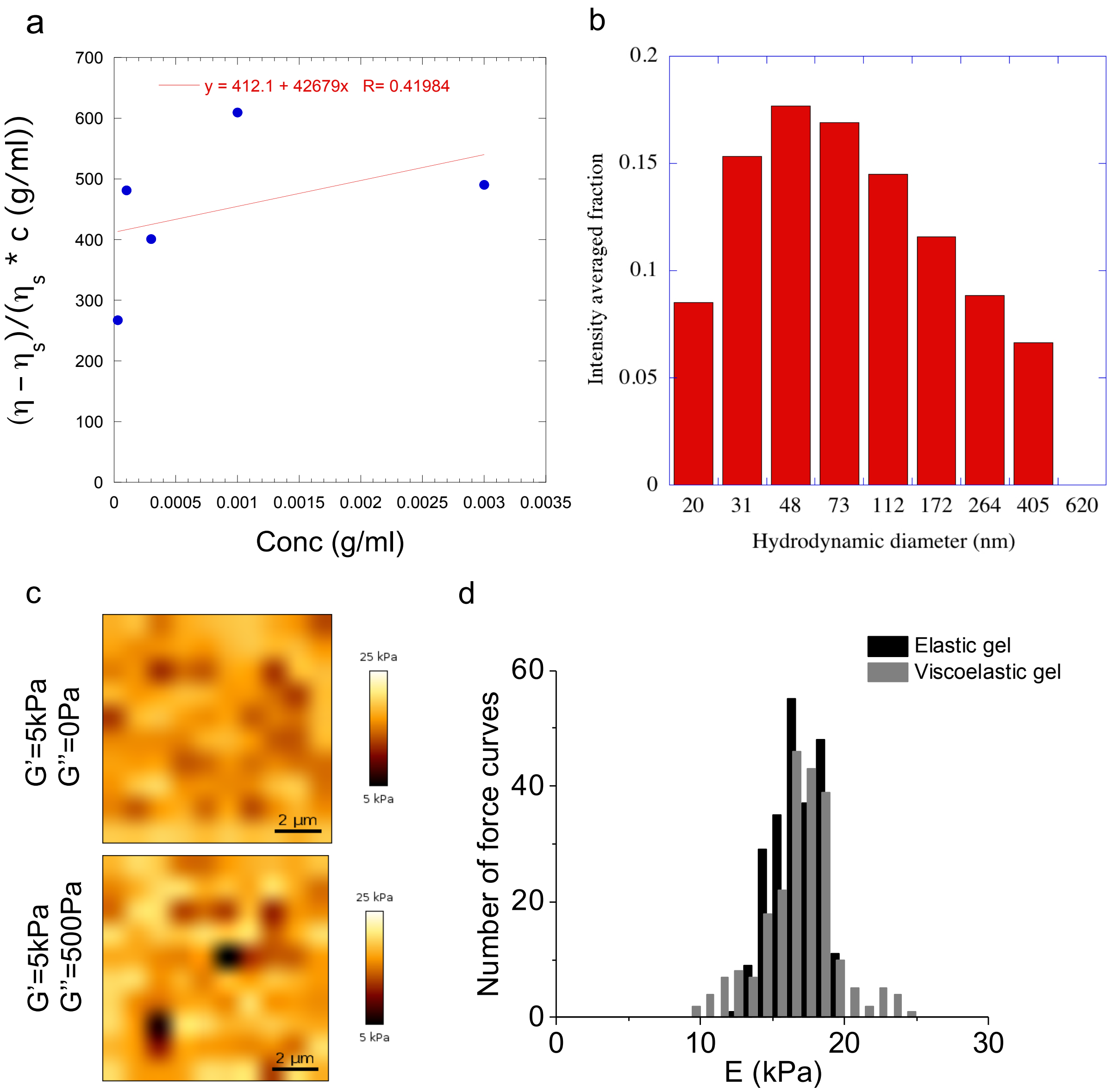

Supplementary Figure 2: Characterization of the linear PAA chains molecular weight and hydrodynamic radius.

a: Relative viscosity of the linear acrylamide solution as a function of the mass concentration of linear PAA.

b: Distribution of hydrodynamic radii of linear PAA in H_2O characterized by dynamic light scattering.

c: AFM stiffness mapping, at 1μm indentation depth, of elastic (top) and viscoelastic (bottom) gels.

d: Spatial distribution of the local Young's modulus as characterized by AFM indentation.

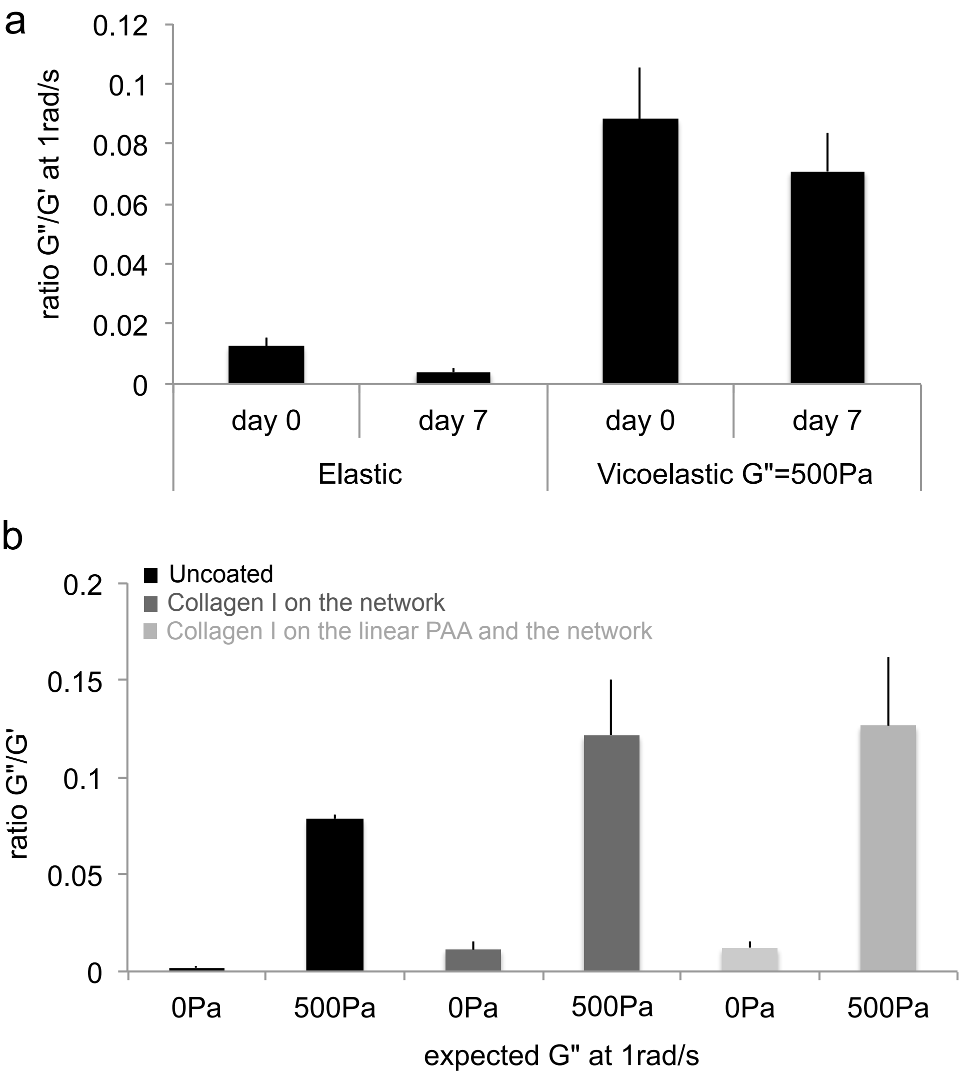

Supplementary Figure 3: Characterization of the gel viscoelasticity evolution over time and of the impact of protein coating I by different methods

a: Evolution of the ratio G"/G' for viscoelastic gels with G”=500 Pa immersed in PBS.

The elastic gels have a very small ratio G”/G’. The ratio G"/G' of the viscoelastic gels decreases approximately 20%, but within the range of error after a 7 day immersion in PBS. n= 5 gels per condition

b: Impact of protein coating on G’ and G” of elastic and viscoelastic gels.

Plot of the ratio G”/G’ for elastic and viscoelastic hydrogels uncoated, and crosslinked to collagen I (50 μg/mL) only on the network of polyacrylamide (NHS) or on both the network and the linear PAA (SS). n= 5 gels per condition. Error bars represent the standard error.

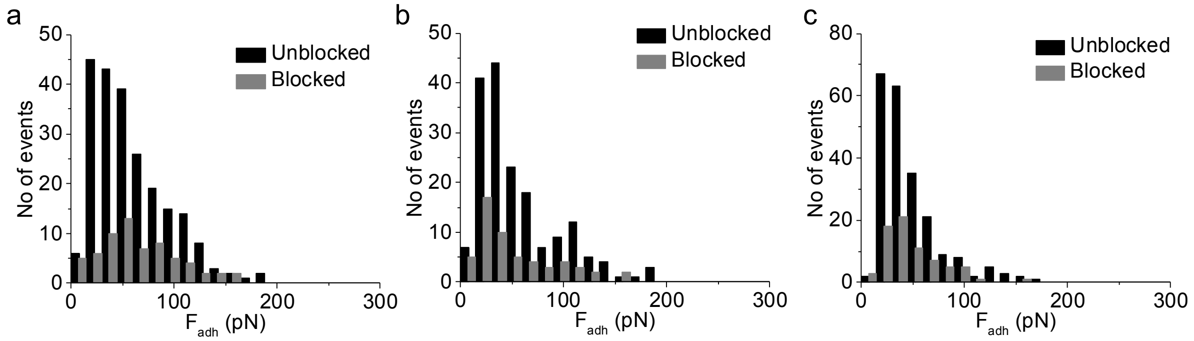

Supplementary Figure 4: Blocking experiments performed with anti-collagen I antibodies, on PAA gels functionalized with collagen I.

a: Blocking experiment performed on an elastic gel presenting collagen I on the network of PAA.

b: Blocking experiment performed on a viscoelastic gel presenting collagen I on the network of PAA.

c: Blocking experiment performed on a viscoelastic gel presenting collagen I on the linear PAA.

Supplementary Methods

Viscometry measurement

The 5% linear acrylamide solution was been diluted with H_2O to obtain 5 solutions of 1.10^{-2} g/L, 3.10^{-2} g/L, 1.10^{-1} g/L, 3.10^{-1} g/L,1 g/L and 3 g/L. The viscosity of each solution was measured with an Ubbelohde viscometer (Cannon Instrument Company). The intrinsic viscosity [η] was calculated from the expression $[\eta]=\text{limit } c\rightarrow 0\ [(\eta-\eta_0)/\eta_0\times c]$ where η, η_0, and c are solution viscosity, solvent viscosity, and concentration of polymer, respectively.

The measured value of [η] was 427 ml/g. The molecular weight, Mw, of the linear PAA was calculated using the Mark-Houwink relation as described by Munk et al. [1]: $[\eta]_{ml/g} = 3.09 \times 10^{-2}\ Mw^{0.67}$. The molecular weight calculated from this relation is 1,600,000 g/mol.

Calculation of hydrodynamic diameter from molecular weight of linear PAA

The radius of gyration Rg of a polyacrylamide chain with a molecular weight of 1,600,000 was calculated assuming 2 flexible C-C bonds each with a length of L=0.154 nm per acrylamide subunit with a Mw per acrylamide subunit = 71 g/mol. The polymer would contain 45,070 subunits and have a contour length of 6940 nm. Making the conservative assumption that polyacrylamide is a random coil, and therefore $Rg^2 = nL^2$ leads to the result that Rg= 33 nm and the diameter is 66 nm. For random coils in a good solvent like water, the chain will be somewhat swollen, so hydrodynamic radius will be even bigger. Rg is for random coils is related to the hydrodynamic radius Rh by Rg/Rh ~ 1.2 (ref [1]) leading to Rh = 27.5 nm or hydrodynamic diameter = 55 nm which is within the range of sizes directly measured by DLS (SI Fig 1B).

Dynamic light scattering

The hydrodynamic diameter of the linear acrylamide molecules was characterized at 25°C by dynamic light scattering at 90° using a DynaPro instrument (ProteinSolutions). The 5% linear acrylamide stock solution was serially diluted up to 300 times to ensure an accurate measurement of the hydrodynamic radius in dilute solution, which was determined from a concentration range in which the measured value of diffusion constant no longer changed with increasing dilution. The average hydrodynamic diameter calculated from the Z-averaged diffusion constant was 40 nm, and the size distribution is shown in Fig. SI 1B.

Atomic force microscopy: stiffness mapping

Stiffness maps of the elastic (G'=5kPa, G''=0Pa) and viscoelastic (G'=5kPa, G''=500Pa) polyacrylamide gels were measured using a NanoWizzard4 AFM microscope (JPK Instruments, Germany) and silicon nitride cantilevers (Novascan Technologies, USA) with a nominal spring

constant of 2.0 – 2.4 N/m with a 25µm diameter bead attached to the tip of the cantilever. Force vs. distance curves were collected within a square of 10 µm × 10 µm and analyzed by the Hertz/Sneddon model using JPK data processing software, assuming that the indentation shape is a hemisphere and the Poisson ratio equals 0.5.

Linear PAA diffusion experiment

Elastic and viscoelastic gels were made on glutaraldehyde coverslips. G' and G" of gels were measured with an RFS3 rheometer (TA Instruments) at 2% strain with an 8 mm parallel plate geometry. Then gels were immersed in PBS, and measured again after 7 days. The excess of water on top of the gel was removed to ensure a good contact between the gel and the upper plate of the rheometer. The error of this measurement is slightly increased by the fact that the geometry used is smaller, and that the gels surface might not be perfectly flat.

Impact of protein coating on the viscoelasticity

Gels were made and functionalized in a rigorously similar manner as described in the method section "*Viscoelastic polyacrylamide gel preparation and protein coating".* After rinsing the protein solution, excess water was removed and the gel was placed under the rheometer. G' and G" of gels were measured with a RFS3 rheometer (TA Instruments) at 2% strain with an 8 mm parallel plate geometry.

Supplementary Reference

1. Munk, P., Aminabhavi, T. M., Williams, P., Hoffman, D. E. & Chmelir, M. Some Solution Properties of Polyacrylamide. *Macromolecules* **13,** 871–876 (1980).
